# Magnetically controlled ferromagnetic swimmers

**DOI:** 10.1038/srep44142

**Published:** 2017-03-09

**Authors:** Joshua K. Hamilton, Peter G. Petrov, C. Peter Winlove, Andrew D. Gilbert, Matthew T. Bryan, Feodor Y. Ogrin

**Affiliations:** 1 College of Engineering, Mathematics and Physical Sciences, University of Exeter, Exeter, UK

## Abstract

Microscopic swimming devices hold promise for radically new applications in lab-on-a-chip and microfluidic technology, diagnostics and drug delivery etc. In this paper, we demonstrate the experimental verification of a new class of autonomous ferromagnetic swimming devices, actuated and controlled solely by an oscillating magnetic field. These devices are based on a pair of interacting ferromagnetic particles of different size and different anisotropic properties joined by an elastic link and actuated by an external time-dependent magnetic field. The net motion is generated through a combination of dipolar interparticle gradient forces, time-dependent torque and hydrodynamic coupling. We investigate the dynamic performance of a prototype (3.6 mm) of the ferromagnetic swimmer in fluids of different viscosity as a function of the external field parameters (frequency and amplitude) and demonstrate stable propulsion over a wide range of Reynolds numbers. We show that the direction of swimming has a dependence on both the frequency and amplitude of the applied external magnetic field, resulting in robust control over the speed and direction of propulsion. This paves the way to fabricating microscale devices for a variety of technological applications requiring reliable actuation and high degree of control.

There have been numerous efforts to produce highly controllable and energy efficient self-propelling systems over length scales from micro- to centimetres as such devices could introduce radically new approaches to technology. Any experimental realization however faces a number of challenges especially at small length scales due to the peculiarities of swimming at low Reynolds number (*Re*) which scales with the size of the swimmer, a situation succinctly summarized in the so-called scallop theorem^1^,^2^,^3^. There have been many elegant models produced to show methods of propelling at low Reynolds numbers^4^,^5^,^6^. In addition, strong Brownian forces at these length scales tend to disorient the swimmer, making its trajectory difficult to control. Another major difficulty is the supply of energy to generate the swimmer’s motion. Different strategies with varying levels of success have been used so far, employing chemical^7^,^8^,^9^,^10^ and magnetic energy^11^,^12^,^13^,^14^,^15^,^16^, as well as light^17^,^18^, electric fields^19^, and ultrasound^20^. The magnetic field is a promising source not only because energy can be supplied remotely, but also because in many cases magnetic interactions help to keep the Brownian disorientation at bay thus offering a mechanism of control. As recently argued by Erb *et al*.^21^, actuating strategies based on the use of magnetic fields offer the highest manipulation forces and torques over device length scales from sub-micrometres to millimetres. Previously, we proposed a novel concept for a swimming device based on a pair of elastically coupled particles of different magnetic susceptibility, which can be controlled by a time-varying magnetic field^22^,^23^,^24^. Here we describe the fabrication of such a swimmer on a millimetre length scale and characterise experimentally its swimming performance over a range of Reynolds numbers. A particular emphasis of this study is to demonstrate robust control over the swimmer’s speed and direction of motion, which makes it a highly versatile magnetically-actuated machine. Such controllability and versatility would make miniaturised (micrometre-sized) versions of these devices attractive for a number of biomedical and technological applications.

Previously, we developed a theoretical model of a magnetic swimmer based on a pair of ferromagnetic particles of different size and different anisotropic properties joined by an elastic element^22^,^23^,^24^, and driven by an external time-dependent magnetic field (Fig. 1(a) shows a schematic of the model). Due to the different anisotropic properties of the two particles, the application of an external magnetic field leads to time varying dipolar gradient force between the particles (resulting in a relative radial motion) as well as time-dependent torque (causing an oscillatory rotational motion of the whole system). The combination of these two interactions modulated by the elastic link binding the particles and the hydrodynamic coupling through the viscous fluid was shown to successfully overcome the limitations set by the scallop theorem and led to self-propulsion at low *Re*. Generally, due to the difference in the hydrodynamic interaction in the first half of the cycle, when the particles are closer to each other, with that in the second half of the cycle, when particles are further away, there is an asymmetry in the time-reversal displacement of the particles leading to an overall translation of the system. The model predicted rich dynamical behaviour including both linear and non-linear swimming trajectories and different propulsion regimes. We now describe the construction of such a device and demonstrate how the speed and direction of motion can be controlled by adjusting the parameters (frequency and amplitude) of the external magnetic field without changing its principal direction, which would offer considerable advantages in technological applications. We also investigate the dependence of the propagation speed as a function of the viscosity of the fluid.

## Results

### Device fabrication

In our device the hard particle is composed of NdFeB which, due to its tetragonal crystal structure, has an extremely high uniaxial anisotropy and exhibits a high resistance to demagnetisation. The second particle is made of Fe wire (99.5% purity), an intrinsically soft ferromagnet that is relatively easy to magnetise and demagnetise in weak magnetic fields.

**Figure 1:**
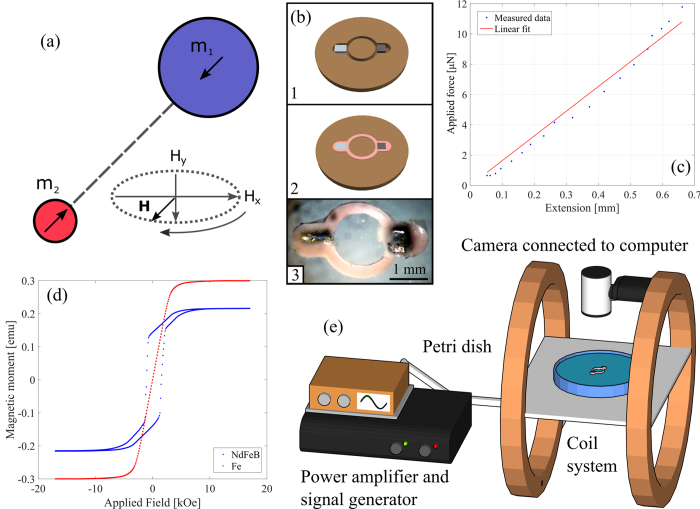
Fabrication and characteristic information. (**a**) Schematic diagram showing the geometry of the modelled ferromagnetic swimmer. The model comprises of two magnetic beads of different size and anisotropic properties connected with an elastic link with zero volume. The larger bead (radius *R*_1_) has a magnetic moment (*m*_1_) that follows the applied magnetic field. The smaller bead (*R*_2_ = *R*_1_/2) has a fixed hard magnetic moment (*m*_2_). **(b)** Fabrication of the device. (1) The particles are placed into a brass mould and aligned using an external magnetic field. (2) Liquid elastomer fills the mould (0.5 mm deep) and is left to cure. (3) The final device. (Dimensions: overall length 3.6 mm, particle edge to edge separation of 1.6 mm (2.2 mm centre to centre), hard particle cubic (0.6 × 0.6 × 0.45 mm), soft particle cylindrical (0.7 mm long, diameter 0.5 mm)). **(c)** Force-extension curve of the silicone rubber link, which is approximately linear over the relevant strain range with an effective spring constant of (1.67 ± 0.08) × 10^−2^ N m^−1^ **(d)** Magnetic hysteresis loops for the hard and soft particles (blue and red, respectively). The magnetic moment of the hard and soft ferromagnetic particles at 2.0 mT are 1.39 × 10^−1^ emu and 2.45 × 10^−2^ emu, respectively. **(e)** The experimental apparatus, consisting of a signal generator and power amplifier (to power the oscillating magnetic field) and a coil system, supplying a field strength of 0.5–2.5 mT. The devices are placed at the fluid-air interface in a Petri dish and observed by a video camera.

The devices (of a typical overall size of about 3.6 mm) were constructed using a mould built in-house as shown in Fig. 1(b) part (1–3). The two ferromagnetic particles were initially fixed with their anisotropy axis along the long axis of the swimmer and then the mould was filled with liquid elastomer. After curing in atmospheric conditions the liquid rubber formed a circular link. Figure 1(b) part (3) shows a typical device. The elastomer encapsulated the magnetic particles and provided the requisite mechanical coupling between them. Several materials were tested and the most satisfactory was found to be silicone rubber (Polycraft), which produced links with the fewest defects (lack of air bubbles) and good adhesion to the metallic particles. The mechanical properties of the formed links were determined using a home-built micromechanical testing apparatus^25^. As illustrated in Fig. 1(c), the force-extension characteristics were linear over the operating range of the device, with an effective spring constant of (1.67 ± 0.08) × 10^−2^ N m^−1^ and negligible hysteresis. Figure 1(d) shows the magnetic hysteresis loops for both the hard and soft particles, demonstrating the expected, and requisite differences in their behaviour.

The swimming capabilities of the devices were studied by examining their mobility in two dimensions at a fluid-air interface. The experimental setup is shown in Fig. 1(e). A 148 mm diameter Petri dish containing water or aqueous sucrose solution is placed within a pair of Helmholtz coils powered with a sinusoidal signal via a standard audio amplifier. The frequency is varied in the range of 20–200 Hz with a magnetic field strength between 0.5 and 2.5 mT. The devices were placed on the fluid-air interface (held there by capillary forces) and observed with a video camera attached to a computer. The device was placed in the centre of the dish which ensured that it was not affected by the curved meniscus near the edges of the Perti dish (the capillary length for water is a few millimetres). Particle tracking software was used to determine both the average speed and direction of migration of the swimmer. A range of sucrose solutions of different kinematic viscosities (from 1 × 10^−6^ to 2.4 × 10^−4^ m^2^ s^−1^) were used to investigate the performance of the swimmers at different *Re*.

### Speed of propagation

**Figure 2:**
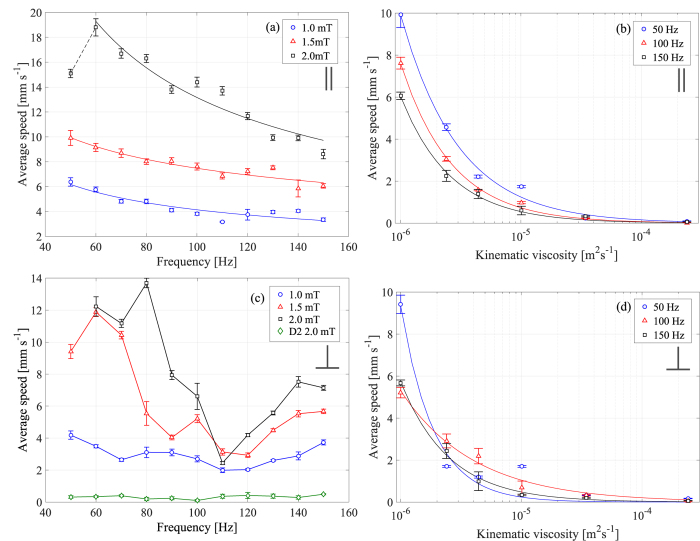
Speed dependencies on external factors. (**a**) Average speed as a function of frequency in water (kinematic viscosity v = 1 × 10^−6^ m^2^ s^−1^) and coil system parallel to the Earth’s magnetic field, at different external magnetic field strengths: 1.0 mT (blue circles), 1.5 mT (red triangles), and 2.0 mT (black squares). The solid lines are lines of best fit (see text). (**b**) Average speed as a function of viscosity (coil system parallel to the Earth’s magnetic field) with an external field strength of 1.5 mT at different frequencies; 50 Hz (blue circles), 100 Hz (red triangles), and 150 Hz (black squares). The lines are best fits. (**c**) Average speed as a function of frequency in water (kinematic viscosity v = 1 × 10^−6^ m^2^ s^−1^) and coil system perpendicular to the Earth’s magnetic field, at different external magnetic field strengths: 1.0 mT (blue circles), 1.5 mT (red triangles), 2.0 mT (black squares) and a device (D2) with an increased particle edge to edge separation of 2.4 mm (3 mm centre to centre) at 2.0 mT (green diamonds). The lines are guides to the eye only. (**d**) Average speed as a function of viscosity (coil system perpendicular to the Earth’s magnetic field) with an external field strength of 1.5 mT at different frequencies; 50 Hz (blue circles), 100 Hz (red triangles), and 150 Hz (black squares). The lines are best fits. The error bars on all plots represent the mean squared error in the speed calculated over a 20 second video at 30 fps.

Systematic investigation of the frequency and viscosity dependences of the average propagation speed has revealed that the orientation of the coil system with respect to the Earth’s magnetic field (~0.02 mT) is an important parameter. Figure 2(a) shows the average translational speed of the device as a function of frequency at three different field strengths when the coils are aligned in parallel to the Earth’s field (i.e. the line joining the geometrical centres of the two coil loops is parallel to the direction of the Earth’s field). A maximum in the frequency dependence is observed at 60 Hz when the field strength is high (Fig. 2(a), 2.0 mT), however in general the speed 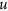 steadily decreases with the increase in frequency, 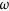 (Fig. 2(a)). Figure 2(b) shows the dependence of the average speed on viscosity, 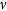, for three different frequencies (again for a parallel alignment). We find a power law dependence, *u* ∝ *ω*^*a*^*ν*^*b*^, with *a* ≈ −0.6 (−0.6 ± 0.2 at 1.0 mT, −0.4 ± 0.1 at 1.5 mT and −0.7 ± 0.2 at 2.0 mT) and *b* close to −1 (−0.9 ± 0.2 at 50 Hz, −1.0 ± 0.1 at 100 Hz and −1.0 ± 0.1 at 150 Hz). These results are in qualitative agreement with our theoretical model^23^ which predicts decreasing speeds with increasing frequency and viscosity (at high enough values of these parameters). Our current simulations of the frequency dependence of the average propagation speed including the effect of a constant bias field (see Supplementary Figure 1(a)) reveal a presence of a maximum at low frequencies, followed by a monotonic decrease of the speed, in similarity to the experimental findings (Fig. 2(a), 2.0 mT). Quantitative differences between theory and experiment are to be expected since the model employs a simplified geometry (two spherical magnetic particles joined by a linear spring of zero volume and placed in the bulk of the fluid). In the current experiments, the shapes of the two particles are not spherical, the swimming occurs at the air-liquid interface and, more importantly, the elastic element is circular, thus likely considerably to affect the fluid flow around the swimmer (a contribution that is neglected in the theoretical model). Given these differences, the qualitative agreement between theory and experiment is a sign of the robustness of the model, which is able to capture the main dynamic effects operating in the system (see also below). The range of Reynolds numbers covered in the experiments (based on the maximum average speed achieved by the swimmers, their overall length and the viscosity of the fluids) was between ~6 × 10^−5^ and ~20 showing that the device is capable of self-propulsion at low to moderate *Re*.

Figure 2(c) and (d) show the same dependences but for a perpendicular orientation of the coil system with respect to the Earth’s magnetic field. This orientation modifies significantly the frequency dependence of the average speed of migration (cf Fig. 2(c) and (a)). In this case the frequency dependence is no longer monotonically decreasing and exhibits more than one maximum even at low field strengths. At higher field strengths, there is a tendency of fast decrease in the speed with increase of frequency up to ca. 110 Hz, after which the speed begins rising again.

It is evident that the presence of a constant bias field (in this case the Earth’s magnetic field) is an important factor that can be used to control the performance of the swimmer. The bias field provides an additional symmetry axis and contributes to the torque affecting the swimmer’s speed. We predict that, depending on the frequency, it also leads to switching between different swimming regimes (characterized by differences in the relative motions of the two magnetic particles, see below), resulting in a complex frequency dependence. This is strongly supported by our current simulations which show similar differences in the frequency dependence between a parallel and a perpendicular bias field (Supplementary Figure 1). For parallel orientation, the theoretical dependence has a maximum at low frequency but is mostly decreasing in the regime of stable swimming (Supplementary Figure 1(a), cf Fig. 2(a)). However, when a perpendicular bias is applied, the theoretical frequency dependence gains two maxima (in qualitative similarity to the experiment, cf Supplementary Figure 1(b) and Fig. 2(b)). Both experiments and simulations reveal rich dynamics which has implications for the control of the swimming motion (speed and direction), as discussed below.

Further experimental characterisation of the performance of the swimmer is given in the Supplementary Information file. Supplementary Figure 2 shows the dependence of the average speed on the external field amplitude at different frequencies (parallel alignment). As expected, the speed increases with the field strength, however at high frequencies (150 Hz) the average speed reaches a saturation at high external field amplitudes.

Small variation in the geometrical parameters of the swimmer (e.g. the equilibrium distance between the magnetic particles) drastically affects the swimming performance due to the strong dependence of the magnetic particle-particle interaction forces on the particle separation. This is illustrated in Fig. 2(c) (green diamonds), where increasing the separation by ~36% reduces the speed by almost two orders of magnitude. The dynamics of the system depends on the gradient forces generated by the magnets, the elasticity of the coupling and thus the strain in the elastic coupling over the oscillation cycle. The magnetic force between the two particles can be estimated if the geometry of the particles is known^26^ (as illustrated in Supplementary Figure 3, where the effect of the external field strength on the particle magnetisation is taken into account as well). Assuming anti-parallel alignment of the magnetisation, an external field of 2.0 mT and an equilibrium inter-particle separation of 2.2 mm (centre to centre), and balancing the magnetic force against the elastic force of the link, we obtain a maximum extension of 0.25 mm (a deformation of ~7% of the length). If we characterise the deformation of the single parameter of longitudinal strain, the theory predicts a deformation of ~13% of the total length. If the equilibrium particle separation is increased by ~36%, as for the device D2 (shown in Fig. 2(c), green diamonds), the resulting extension decreases to 0.13 mm, which ultimately results in reduced speed of motion.

### Directional control of the device

**Figure 3:**
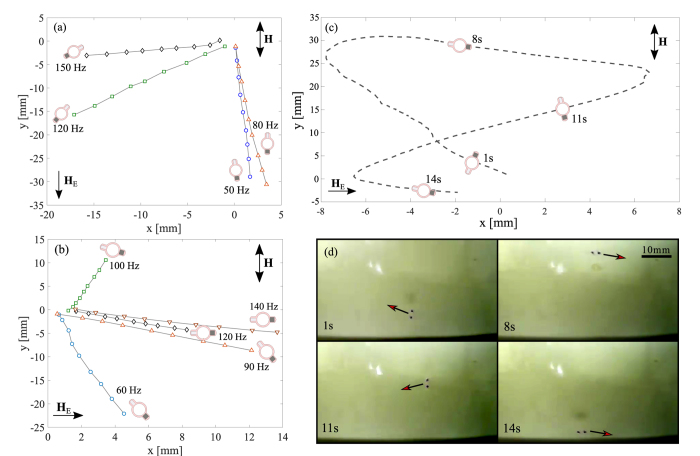
Directional control using external factors. Effects of the frequency and amplitude of the applied field on the direction of migration. The direction of the applied magnetic field, **H**, and the Earth’s magnetic field, **H**_**E**_ (~0.02 mT) are indicated. (**a**) Direction of motion as a function of frequency at 2.0 mT for a parallel alignment between **H** and **H**_**E**_. The mean orientation of the swimmer is shown schematically for each frequency. The final point on each trajectory is at 1.9 seconds. (**b**) Direction of motion as a function of frequency at 2.0 mT for a perpendicular alignment between **H** and **H**_**E**_. The mean orientation of the swimmer is shown schematically for each frequency. The final point on each trajectory is at 1.9 seconds. (**c**) A figure of eight trajectory produced by varying both the frequency and amplitude (Supplementary Movie 1). The four sketches of the device show its orientation at the respective time points. (**d**) Still frames showing the direction of propagation at four different time points.

One of the main advantages of the swimmer presented here is that its direction of motion can be easily controlled by adjusting the frequency and amplitude of the applied external magnetic field, without spatial repositioning of the coil system. Figure 3(a) and (b) illustrate in detail the effect of the frequency of the applied field on the orientation and trajectory of the device. In the parallel alignment between the external field and the Earth magnetic field (Fig. 3(a)), we were able to control the direction of propagation within about 95° by adjusting the frequency (see the trajectories for 50 Hz and 150 Hz). The large jump in direction between 80 Hz and 120 Hz is due to the transition between different swimming regimes caused by critical frequencies. When the two fields were aligned perpendicularly to each other, the swimmer tended to migrate to the right (Fig. 3(b)), but again the direction of motion was easily controlled by changing the frequency. In this case, we achieved a slightly wider coverage of the space (ca. 115°) as can be seen from Fig. 3(b) by comparing the trajectories of the swimmer for 60 Hz and 100 Hz.

The amplitude of the external magnetic field is another parameter that can be used to bring an additional degree of control over the direction of swimming. When the frequency and amplitude are adjusted simultaneously, one can achieve virtually any trajectory. This is demonstrated in Fig. 3(c) which shows a high degree of control achieved by only varying the frequency and amplitude within the ranges 50–150 Hz and 0.5–2.0 mT, respectively (see also Supplementary Movie 1). The position, orientation and the instantaneous direction of motion at four time points are illustrated in the snapshots of Fig. 3(d), and indicated in Fig. 3(c) for clarity. In this example the average speed along the trajectory is 5.6 mm s^−1^. To further illustrate the control of the device, Supplementary Figure 4 shows a trajectory plot in which both frequency and amplitude have been varied, when the system has perpendicular alignment with the Earth’s magnetic field.

It is important to note that the swimmer is able to propagate by employing different swimming regimes. In some cases, the device propagates in the direction of its primary axis (i.e. the line joining the two magnetic particles, see e.g. Fig. 3(a) at 50 Hz and Fig. 3(c) at 8 s). For other values of the parameters (frequency and amplitude) we observe motion in a direction perpendicular to the principal axis of the swimmer (e.g. Fig. 3(b) at 100 Hz and Fig. 3(c) at 11 s). Our theoretical model^23^ was able to predict the existence of these two propulsion regimes in which the direction of motion is either along or perpendicular to the main axis of the swimmer (cf ref. 23 Figs 2(e) and 3(e)). The modelling predicts that when the swimmer moves along its principal axis, the individual magnetic particles exhibit large amplitude undulations, the internal motion resembling a ‘pendulum’ (see Supplementary Movie 2). In contrast, when the swimmer moves perpendicular to its primary axis, it mainly rocks about a mean angle (see Supplementary Movie 3). Furthermore, current simulations predict our experimental findings that the propulsion direction can be controlled by frequency (Supplementary Figure 5, cf Fig. 3(b)) and that the propulsion regime is also frequency dependent.

The results presented here demonstrate how a high level of control over the speed and direction of propagation can easily be achieved by tuning the frequency and amplitude of the external magnetic field and by adding a very small constant bias to it (in this case the Earth’s field, ~0.02 mT). This would be highly advantageous in technological applications requiring robust actuation and control.

## Conclusions

In summary, we have described the fabrication of a novel autonomous swimming device, based on magnetic, elastic and hydrodynamic interactions of a pair of particles (a hard NdFeB magnet and a soft Fe magnet), controlled by an external oscillating magnetic field. We investigated the swimmer’s response to changes in the external magnetic field (frequency and amplitude) as well as fluid viscosity and demonstrated how its speed and direction of motion can be controlled using the magnetic field parameters. We showed that inclusion of a small constant bias to the external field could be advantageous for establishing tighter control over both the direction of motion of the device and its speed. This device is easily scalable to sizes of tens of micrometres and, given its ability to swim at low Reynolds numbers whilst lending itself to robust control, could potentially be used in a wide range of tasks such as transport in microfluidic and lab-on-a-chip devices or novel targeted drug delivery systems.

## Materials and Method

### Materials and Device Fabrication

NdFeB magnets (5 × 2 × 0.45 mm) and 99.5% pure Fe wire (diameter 0.5 mm) were purchased from First4Magnets and Sigma-Aldrich, respectively. The materials were cut using a diamond dicer (LoadPoint Micro Ace 3 Dicing Saw) to produce the hard (0.6 × 0.6 × 0.45 mm) and soft (0.7 mm long with diameter 0.5 mm) particles. Sucrose (reagent grade) and sodium azide were purchased from Sigma-Aldrich and were dissolved in water using a heating magnetic stirrer to produce sucrose solutions with concentrations of 30% (v = 2.48 × 10^−6^ m^2^ s^−1^), 40% (v = 4.41 × 10^−6^ m^2^ s^−1^), 50% (v = 1.01 × 10^−5^ m^2^ s^−1^), 60% (v = 3.45 × 10^−5^ m^2^ s^−1^), and 70% (v = 2.4 × 10^−4^ m^2^ s^−1^). Sodium azide (30 mg per 100 ml) was added to prevent bacterial growth. Polycraft silicone rubber and fast cure catalyst (GP-3481-F) were purchased from MBFibreglass. The two components were mixed with a weight ratio of 1:10 (catalyst:silicone) and placed into the brass mould (produced in-house) with the two particles (a constant magnetic field is used to align the direction of the hard particle) and cured at room temperature for 6 hours. The moulds produced devices of link diameter of 2 mm and total length 3.6 mm. 6 devices were produced with a particle separation of 2.2 mm, and 4 with the increased particle separation (3 mm). Other elastomers were also tested. Dow Corning 781 silicone sealant cured at room temperature for 4 hours produced a large number of air bubble defects. Liquid latex (source) cured at room temperature for 6 hours also produced a device with a large number of visible defects.

### Measurement of Migration Characteristics

Each coils of the magnetic system had approximately 560 turns with a width of 61 mm, inner bobbin diameter 155 mm and height 37 mm.

The devices were placed at the fluid-air interface of the sucrose solution contained in a 148 mm diameter Petri dish and positioned in the centre of the coil system. The oscillating magnetic field was applied and the motion of the swimmer was observed with a video camera attached to computer. Particle tracking software (Open Source software - Tracker) was used to determine both the average speed and direction of propagation of the swimmer. The videos are of length 20 seconds (at 30 fps) and the speed was measured between every frame, and the mean squared error was taken.

### Magnetic hysteresis and elasticity measurements

The magnetic hysteresis loops of the individual particles were obtained using a vibrating sample magnetometer (MicroSense), probing between ±17 kOe, for both the NdFeB and Fe particles. The mechanical properties of the formed links were determined by mounting them in a home-built micromechanical testing apparatus.

### Data Availability

All data created during this research are openly available from the University of Exeter’s institutional repository at https://ore.exeter.ac.uk/repository/handle/XXXXX/YYYYY).

## Additional Information

**How to cite this article:** Hamilton, J. K. *et al*. Magnetically controlled ferromagnetic swimmers. *Sci. Rep.* **7**, 44142; doi: 10.1038/srep44142 (2017).

**Publisher's note:** Springer Nature remains neutral with regard to jurisdictional claims in published maps and institutional affiliations.

## Supplementary Material

Supplementary Information

Supplementary Movie 1

Supplementary Movie 2

Supplementary Movie 3
