# Supplementary material for: Magnetically controlled ferromagnetic swimmers
Source: Sci Rep. 2017 Mar 9;7:44142. doi: 10.1038/srep44142 (PMC5343437; doi:10.1038/srep44142)
Supplement: Supplementary Information [file srep44142-s1.pdf]

Supplementary Information

Magnetically Controlled Ferromagnetic Swimmers

Joshua K. Hamilton[1], Peter G. Petrov[1], C. Peter Winlove[1], Andrew D. Gilbert[1], Matthew T. Bryan[1], and Feodor Y. Ogrin[1,*]

[1]College of Engineering, Mathematics and Physical Sciences, University of Exeter, Exeter, UK

*Author for correspondence: Feodor Ogrin, University of Exeter, Physics Building, Stocker Road, Exeter, EX4 4QL, UK. F.Y.Ogrin@exeter.ac.uk

Movie captions

Supplementary Movie 1: Real time video showing the trajectory of the device performing a figure of eight. This was achieved only by varying the frequency and amplitude of the applied field. The device is on the fluid-air interface.

Supplementary Movie 2: Real time video showing the propulsion of the device in a magnetic field of 2.5 mT oscillating at 50 Hz. In this case the experimental setup is parallel to the Earth's magnetic field. This demonstrates the swimming regime in which the device is migrating along its principal axis (i.e. the line joining the two magnetic particles).

Supplementary Movie 3: Real time video, showing the propulsion of the device in a magnetic field of 0.9 mT oscillating at 150 Hz. In this case the experimental setup is parallel to the Earth's magnetic field. This demonstrates another swimming regime, during which the device is migrating in a direction perpendicular to its principal axis.

Supplementary figures

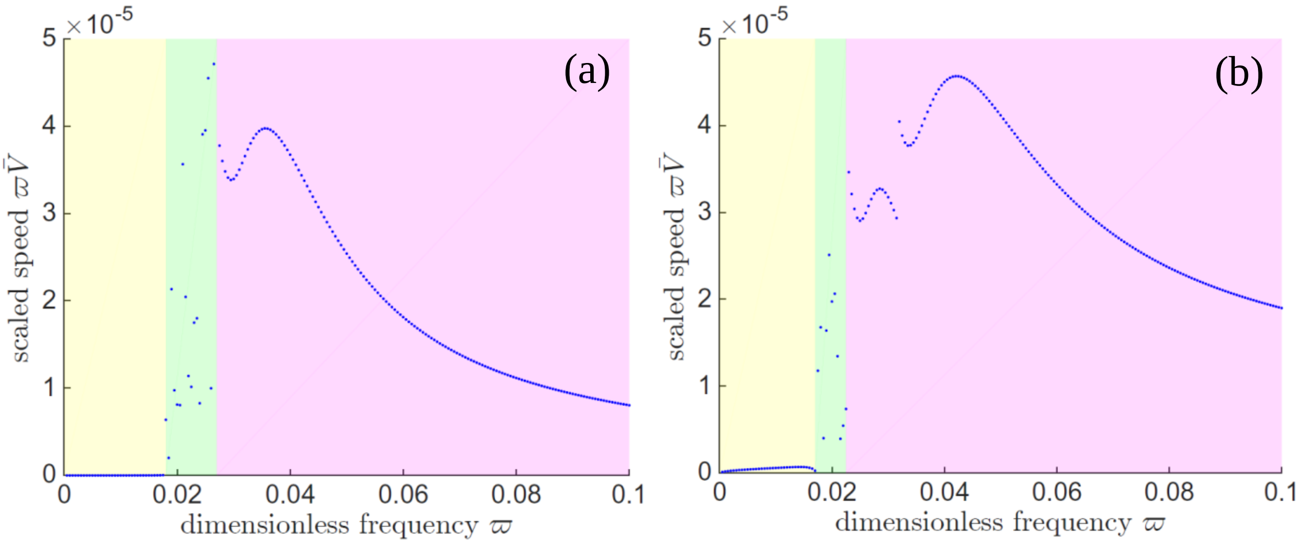

Supplementary Figure 1. Simulated dependencies of the average speed of the swimmer on frequency (dimensionless) for a parallel (**a**) and perpendicular (**b**) bias field. Three distinct swimming behaviours are observed (depicted in different colours): no or very slow swimming (yellow), incoherent swimming (green) and stable swimming (purple). The externally applied oscillating field is almost uniaxial (1% aspect ratio) to mimic the uniaxial field in experiment and the bias field is 0.5% of the maximum external field. The rest of the parameters are the same as in Ref. [23]. Initially, the swimmer is aligned with its primary axis along the bias field as in the experiments.

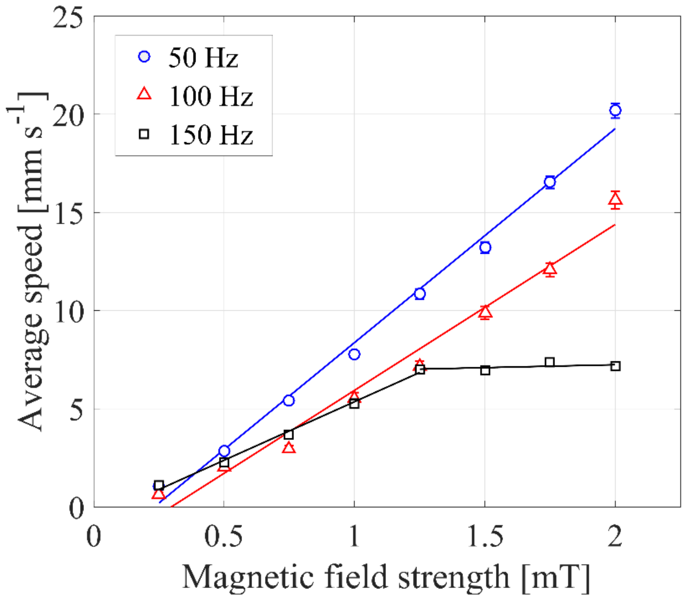

Supplementary Figure 2. Dependence of the average speed of the device (in water) on the external field amplitude at different frequencies: 50 Hz (dark blue circle), 100 Hz (red triangle), and 150 Hz (black square).

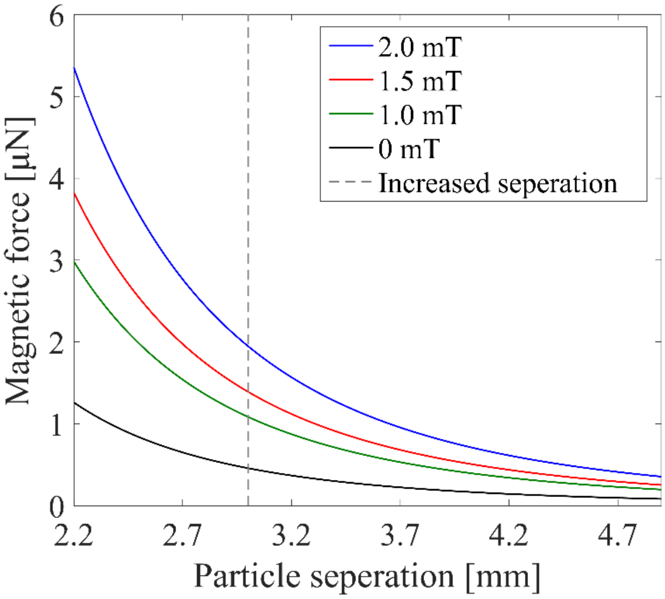

Supplementary Figure 3. Analytical estimate of the magnetic force between the hard and soft ferromagnetic particles as a function of the separation between them in the presence of external magnetic fields of different strength (green: 1.0 mT, red: 1.5 mT and blue: 2.0 mT) and zero external magnetic field (black). The vertical dashed line corresponds to increased particle separation of ~ 36%, as for the device D2 (shown in Fig. 2(c), green diamonds).

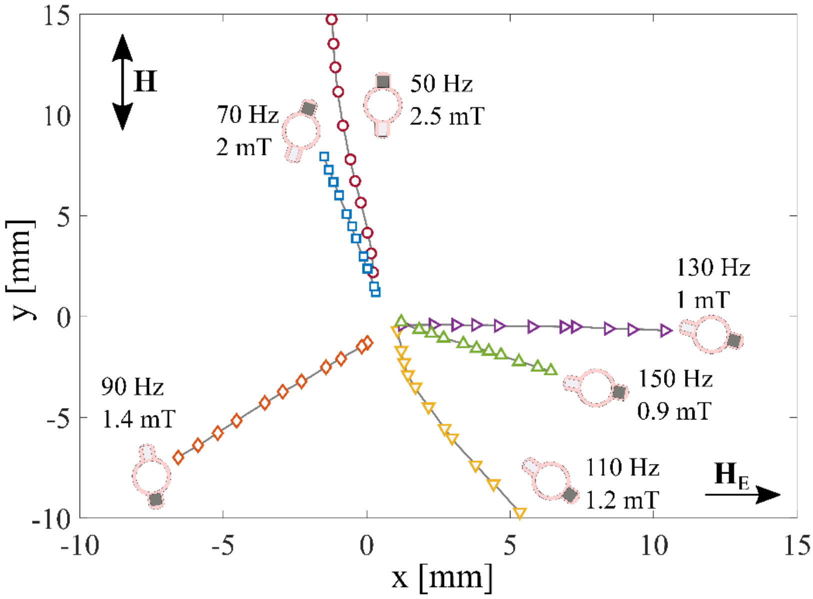

Supplementary Figure 4. Direction of motion as a function of different frequencies and magnetic field strengths (ranging between 50 – 150 Hz and 2.5 – 0.9 mT, respectively) for a perpendicular alignment between **H** and **H**$_\mathbf{E}$. The mean orientation of the swimmer is shown schematically for each frequency. The final point on each trajectory is at 2.7 seconds.

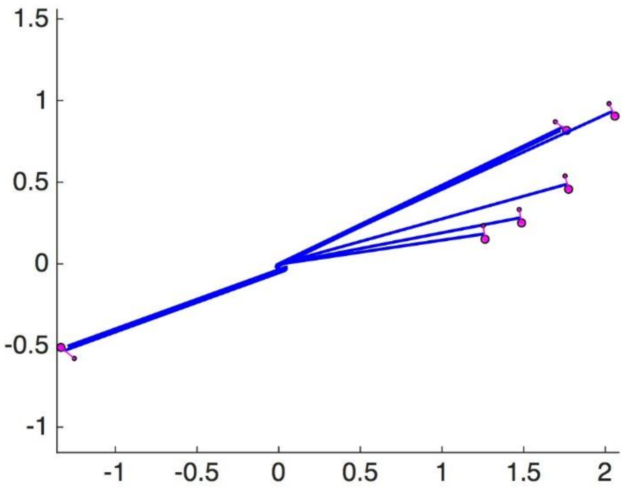

Supplementary Figure 5. Simulated trajectories at different frequencies. Six trajectories are shown obtained by varying the dimensionless frequency between 0.025 and 0.075. The parameters used are the same as in Supporting Figure 2. The mean orientation of the swimmer is shown schematically for each trajectory.
